# Supplementary material for: Identification of Sugarcane Host Factors Interacting with the 6K2 Protein of the Sugarcane Mosaic Virus
Source: Int J Mol Sci. 2019 Aug 8;20(16):3867. doi: 10.3390/ijms20163867 (PMC6719097; doi:10.3390/ijms20163867)
Supplement: Supplementary file 1 [file ijms-20-03867-s001.pdf]

International Journal of
Molecular Sciences

MDPI

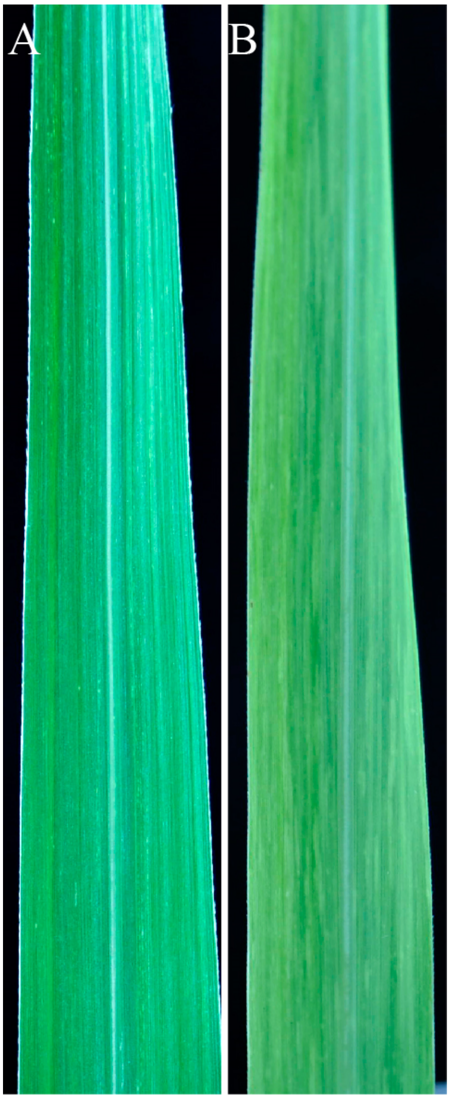

Figure 1. Mosaic symptom on the leaves of sugarcane cultivar ROC22. The healthy leaf was shown in (**A**) and the mosaic leaf was shown in (**B**).

Table S1. Primers used in this study.

Genes	Primers	Primer sequences*	Strategy/Constructs
SCMV-6K2	6K2-F	5′-ATGGGAATGGATGCAACAGC-3′	pPOTO-6K2
	6K2-R	5′-CCCCTGGTGCGACACGTTTGTGC-3′	
SCMV-6K2	pBT3-STE-6K2-F	5′-ATTACCAAGGCCATTACGGCCATGGGAATGGATGCAACAGC-3′	pBT3-STE-6K2
	pBT3-STE-6K2-R	5′-AACTGATTGGCCGAGGCGGCCCCCTGGTGCGACACGTTTGTGC-3′	
SCMV-6K2	Gateway-6K2-F	5′-GGGGACAAGTTTGTACAAAAAAGCAGGCTTCATGGAATGGATGCAACAG-3′	pEarleyGate102-6K2
	Gateway-6K2-R	5′- GGGGACCACTTTGTACAAGAAAGCTGGGTCTGGTGCGACACGTTTGTGC-3′	
	pPR3-N-F	5′-GTCGAAAATTCAAGACAAGG-3′	Yeast plasmid sequencing
	pPR3-N-R	5′-AAGCGTGACATAACTAATTAC-3′	
ScPsbS	ScPsbS-F	5′-ATGGCGCAGTCCATGCTCAT-3′	pPOTO-ScPsbS
	ScPsbS-R	5′-CTACTCTTCGTCCTCGCTGA-3′	
ScPsbS	pPR3-N-ScPsbS-F	5′-ATTAACAAGGCCATTACGGCCATGGCGCAGTCCATGCTCAT-3′	pPR3-N-ScPsbS
	pPR3-N-ScPsbS-R	5′-TTGACTAAGGCCGAGGCGGCCGCTACTCTTCGTCCTCGCTGA-3′	
ScTMEM208	ScTMEM208	5′-ATGGCGAAGCAGGGGGCGAA-3′	pPOTO-ScTMEM208
	ScTMEM208	5′-TCAGCGAGTCCTAGTTTTGA-3′	
ScTMEM208	pPR3-N-ScTMEM208-F	5′-ATTAACAAGGCCATTACGGCCATGGCGAAGCAGGGGGCGAA-3′	pPR3-N-ScTMEM208
	pPR3-N-ScTMEM208-R	5′-TTGACTAAGGCCGAGGCGGCCGTCAGCGAGTCCTAGTTTTGA-3′	
ScPIP1;2	ScPIP1;2-F	5′-ATGGAGGGGAAGGAGGAGGA-3′	pPOTO-ScPIP1;2
	ScPIP1;2-R	5′-TTAAGACCTGCTCTTGAAC-3′	
ScPIP1;2	pPR3-N-ScPIP1;2-F	5′-ATTAACAAGGCCATTACGGCCATGGAGGGGAAGGAGGAGGA-3′	pPR3-N-ScPIP1;2
	pPR3-N-ScPIP1;2-R	5′-TTGACTAAGGCCGAGGCGGCCGTTAAGACCTGCTCTTGAAC-3′	
ScPsbR	ScPsbR-F	5′-ATGGCGGCCTCCATGATCTCGT-3′	pPOTO-ScPsbR
	ScPsbR-R	5′-TTAGCCGGAGAGGGCGCTG-3′	

ScPsbR	pPR3-N-ScPsbR-F	5′-ATTAACAAGGCCATTACGGCCATGGCGGCCTCCATGATCTCGT-3′	pPR3-N-ScPsbR
	pPR3-N-ScPsbR-R	5′-TTGACTAAGGCCGAGGCGGCCGTTAGCCGGAGAGGGCGCTG-3′	
ScVAMP727	ScVAMP727-F	5′-ATGATGAACAGCAGCAAGCAG-3′	pPOTO-ScVAMP727
	ScVAMP727-R	5′-CTAACATTTGAATCCCCTGCA-3′	
ScVAMP727	pPR3-N-ScVAMP727-F	5′-ATTAACAAGGCCATTACGGCCATGATGAACAGCAGCAAGCAG-3′	pPR3-N-ScVAMP727
	pPR3-N-ScVAMP727-R	5′-TTGACTAAGGCCGAGGCGGCCGCTAACATTTGAATCCCCTGCA-3′	
ScVHA-C	ScVHA-C-F	5′-ATGTCGTCGGTATTCAGCG-3′	pPOTO-ScVHA-C
	ScVHA-C-R	5′-CTAATCTGCACGGGATTGG-3′	
ScVHA-C	pPR3-N-ScVHA-C-F	5′-ATTAACAAGGCCATTACGGCCATGTCGTCGGTATTCAGCG-3′	pPR3-N-ScVHA-C
	pPR3-N-ScVHA-C-R	5′-TTGACTAAGGCCGAGGCGGCCGCTAATCTGCACGGGATTGG-3′	
ScPIP2;7	ScPIP2;7-F	5′-ATGTCGAAGGATGACGTGGC-3′	pPOTO-ScPIP2;7
	ScPIP2;7-R	5′-TCACACCGTGGCGCTGGTGC-3′	
ScPIP2;7	pPR3-N-ScPIP2;7-F	5′-ATTAACAAGGCCATTACGGCCATGTCGAAGGATGACGTGGC-3′	pPR3-N-SCcPIP2;7
	pPR3-N-ScPIP2;7-R	5′-TTGACTAAGGCCGAGGCGGCCGTCACACCGTGGCGCTGGTGC-3′	
ScBGLU31	ScBGLU31-F	5′-ATGGTGGAGCTGACACCGCGTG-3′	pPOTO-ScBGLU31
	ScBGLU31-R	5′-TCACTCCGAATAGGTGGAGC-3′	
ScBGLU31	pPR3-N-ScBGLU31-F	5′-ATTAACAAGGCCATTACGGCCATGGTGGAGCTGACACCGCGTG-3′	pPR3-N-ScBGLU31
	pPR3-N-ScBGLU31-F	5′-TTGACTAAGGCCGAGGCGGCCGTCACTCCGAATAGGTGGAGC-3′	
ScGONST4	ScGONST4-F	5′-ATGGCAAAGCAGTATTACG-3′	pPOTO-ScGONST4
	ScGONST4-R	5′-TTAGGTTGTTCCATCAGGCT-3′	
ScGONST4	pPR3-N-ScGONST4-F	5′-ATTAACAAGGCCATTACGGCCATGGCAAAGCAGTATTACG-3′	pPR3-N-ScGONST4
	pPR3-N-ScGONST4-R	5′-TTGACTAAGGCCGAGGCGGCCGTTAGGTTGTTCCATCAGGCT-3′	
ScPMP22	ScPMP22-F	5′-ATGGCGGCGGCGTCGGCGTC-3′	pPOTO-ScPMP22
	ScPMP22-R	5′-CTATGTGGAGTTGTCCTTTG-3′	
ScPMP22	pPR3-N-ScPMP22-F	5′-ATTAACAAGGCCATTACGGCCATGGCGGCGGCGTCGGCGTC-3′	pPR3-N-ScPMP22
	pPR3-N-ScPMP22-R	5′-TTGACTAAGGCCGAGGCGGCCGCTATGTGGAGTTGTCCTTTG-3′	

ScVTE3	ScVTE3-F	5′-ATGCCCTCCACCTACGCGC-3′	pPOTO-ScVTE3
	ScVTE3-R	5′-TCAGATTGGCATGCCTTTTG-3′	
ScVTE3	pPR3-N-ScVTE3-F	5′-ATTAACAAGGCCATTACGGCCATGCCCTCCACCTACGCGC-3′	pPR3-N-ScVTE3
	pPR3-N-ScVTE3-R	5′-TTGACTAAGGCCGAGGCGGCCGTCAGATTGGCATGCCTTTTG-3′	
ScZIFL1	ScZIFL1-F	5′-ATGGCCGGCGGTGGCGGCGA-3′	pPOTO-ScZIFL1
	ScZIFL1-R	5′-TTATGAATGCCTCATTGAAT-3′	
ScZIFL1	pPR3-N-ScZIFL1-F	5′-ATTAACAAGGCCATTACGGCCATGGCCGGCGGTGGCGGCGA-3′	pPR3-N-ScZIFL1
	pPR3-N-ScZIFL1-R	5′-TTGACTAAGGCCGAGGCGGCCGTTATGAATGCCTCATTGAAT-3′	
ScTIP1;2	ScTIP1;2-F	5′-ATGCCGGTGAGCAGGATCG-3′	pPOTO-ScScTIP1;2
	ScTIP1;2-R	5′-TCAGTAGTCTGCGGTGGGCA-3′	
ScTIP1;2	pPR3-N-ScTIP1;2-F	5′-ATTAACAAGGCCATTACGGCCATGCCGGTGAGCAGGATCG-3′	pPR3-N-ScTIP1;2
	pPR3-N-ScTIP1;2-R	5′-TTGACTAAGGCCGAGGCGGCCGTCAGTAGTCTGCGGTGGGCA-3′	
ScTPT	ScTPT-F	5′-ATGTCGGCGCTCGGAACGCT-3′	pPOTO-ScTPT
	ScTPT-R	5′-TCATGCGCTCTTTTTCCTTT-3′	
ScTPT	pPR3-N-ScTPT-F	5′-ATTAACAAGGCCATTACGGCCATGTCGGCGCTCGGAACGCT-3′	pPR3-N-ScTPT
	pPR3-N-ScTPT-R	5′-TTGACTAAGGCCGAGGCGGCCGTCATGCGCTCTTTTTCCTTT-3′	
ScRNS4	ScRNS4 -F	5′-ATGGGAGGCCGCAGAACAAT-3′	pPOTO-ScRNS4
	ScRNS4 -R	5′-TGCTCCCCACTCAGACCATG-3′	
ScRNS4	pPR3-N-ScRNS4 -F	5′-ATTAACAAGGCCATTACGGCCATGGGAGGCCGCAGAACAAT-3′	pPR3-N-ScRNS4
	pPR3-N-ScRNS4 -R	5′-TTGACTAAGGCCGAGGCGGCCGTGCTCCCCACTCAGACCATG-3′	
ScSULTR3-3	ScSULTR3-3-F	5′-ATGGTGGGTATGAGAGGTGC-3′	pPOTO-ScSULTR3-3
	ScSULTR3-3-R	5′-TTAGGGTTTTGTCATCTTGG-3′	
ScSULTR3-3	pPR3-N-ScSULTR3-3-F	5′-ATTAACAAGGCCATTACGGCCATGGTGGGTATGAGAGGTGC-3′	pPR3-N-ScSULTR3-3
	pPR3-N-ScSULTR3-3-R	5′-TTGACTAAGGCCGAGGCGGCCGTTAGGGTTTTGTCATCTTGG-3′	
ScPPT2	ScPPT2-F	5′-ATGCAGAGCGCCGCGGCTCT-3′	pPOTO-ScPPT2
	ScPPT2-R	5′-TTAAGAAGTCTTCGGCTTGG-3′	

ScPPT2	pPR3-N-ScPPT2-F	5′-ATTAACAAGGCCATTACGGCCATGCAGAGCGCCGCGGCTCT-3′	pPR3-N-ScPPT2
	pPR3-N-ScPPT2-R	5′-TTGACTAAGGCCGAGGCGGCCGTTAAGAAGTCTTCGGCTTGG-3′	
ScCAB1	ScCAB1-F	5′-ATGCAGAGCGCCGCGGCTCT-3′	pPOTO-ScCAB1
	ScCAB1-R	5′-TCACTTGCCGGGCACGAAGT-3′	
ScCAB1	pPR3-N-ScCAB1-F	5′-ATTAACAAGGCCATTACGGCCATGCAGAGCGCCGCGGCTCT-3′	pPR3-N-ScCAB1
	pPR3-N-ScCAB1-R	5′-TTGACTAAGGCCGAGGCGGCCGTCACTTGCCGGGCACGAAGT-3′	
ScNCX	ScNCX-F	5′-ATGTCGACTCGCCGGCAGCA-3′	pPOTO-ScNCX
	ScNCX-R	5′-CTATGACCAGCCAAAGACAT-3′	
ScNCX	pPR3-N-ScNCX-F	5′-ATTAACAAGGCCATTACGGCCATGTCGACTCGCCGGCAGCA-3′	pPR3-N-ScNCX
	pPR3-N-ScNCX-R	5′-TTGACTAAGGCCGAGGCGGCCGCTATGACCAGCCAAAGACAT-3′	
ScSERINC3	ScSERINC3-F	5′-ATGTGGGCGGCGTCGTGCCT-3′	pPOTO-ScSERINC3
	ScSERINC3-R	5′-CTAGAATTCCCTGTCAGGAA-3′	
ScSERINC3	pPR3-N-ScSERINC3-F	5′-ATTAACAAGGCCATTACGGCCATGTGGGCGGCGTCGTGCCT-3′	pPR3-N-ScSERINC3
	pPR3-N-ScSERINC3-R	5′-TTGACTAAGGCCGAGGCGGCCGCTAGAATTCCCTGTCAGGAA-3′	
ScULP5	ScULP5-F	5′-ATGATCGAGGTGGTGCTGAACGA-3′	pPOTO-ScULP5
	ScULP5-R	5′-CTAGTTGTAGTAGAGCTCGAGCC-3′	
ScULP5	pPR3-N-ScULP5-F	5′-ATTAACAAGGCCATTACGGCCATGATCGAGGTGGTGCTGAACGA-3′	pPR3-N-ScULP5
	pPR3-N-ScULP5-R	5′-TTGACTAAGGCCGAGGCGGCCGCTAGTTGTAGTAGAGCTCGAGCC-3′	
ScHTA2	ScHTA2-F	5′-ATGGCCGGGAGAGGTAAGGCGATC-3′	pPOTO-ScHTA2
	ScHTA2-R	5′-CTAGTCCTCATCGCCAGCCGCGGCCTT-3′	
ScHTA2	pPR3-N-ScHTA2-F	5′-ATTAACAAGGCCATTACGGCCATGGCCGGGAGAGGTAAGGCGATC-3′	pPR3-N-ScHTA2
	pPR3-N-ScHTA2-R	5′-TTGACTAAGGCCGAGGCGGCCGCTAGTCCTCATCGCCAGCCGCGGCCTT-3′	
ScTSPAN18	ScTSPAN18-F	5′-ATGGCGCGTCGCCGGAGCAG-3′	pPOTO-ScTSPAN18
	ScTSPAN18-R	5′-TCACTGAGACGGTGCTGCAT-3′	
ScTSPAN18	pPR3-N-ScTSPAN18-F	5′-ATTAACAAGGCCATTACGGCCATGGCGCGTCGCCGGAGCAG-3′	pPR3-N-ScTSPAN18
	pPR3-N-ScTSPAN18-R	5′-TTGACTAAGGCCGAGGCGGCCGTCACTGAGACGGTGCTGCAT-3′	

ScHSP82	ScHSP82-F	5′-ATGGCCTCGGAGACGGAGAC-3′	pPOTO-ScHSP82
	ScHSP82-R	5′-TTAGTCGACCTCCTCCATC-3′	
ScHSP82	pPR3-N-ScHSP82-F	5′-ATTAACAAGGCCATTACGGCCATGGCCTCGGAGACGGAGAC-3′	pPR3-N-ScHSP82
	pPR3-N-ScHSP82-R	5′-TTGACTAAGGCCGAGGCGGCCGTTAGTCGACCTCCTCCATCT-3′	
